# Synchronized Tick Population Oscillations Driven by Host Mobility and Spatially Heterogeneous Developmental Delays Combined

**DOI:** 10.1007/s11538-021-00874-8

**Published:** 2021-04-18

**Authors:** Xue Zhang, Jianhong Wu

**Affiliations:** 1 College of Science, Northeastern University, Shenyang, 110819 Liaoning China; 2 Laboratory for Industrial and Applied Mathematics, York University, Toronto, ON M3J 1P3 Canada

**Keywords:** Patchy models, Delay equations, Synchronized oscillations, Diapause, Tick population dynamics, Tick-borne disease dynamics, MSC 92D25, MSC 35D30

## Abstract

We consider a coupled system of delay differential equations for a single-species tick population dynamics, assuming feeding adult ticks are distributed by their hosts in a spatially heterogeneous environment consisting of two patches where egg ticks produced will complete their life cycles with different, normal and diapause, developmental delays. We show that the mobility of adult tick host and the diapause developmental delay combined drive a synchronized oscillation in the total tick populations around a uniquely defined positive equilibrium, and this synchronization makes the oscillatory patterns much simpler in comparison with multi-peak oscillations exhibited in the absence of host mobility.

## Introduction

Tick-borne diseases, including tick-borne encephalitis, Lyme disease, anaplasmosis and Crimean–Congo hemorrhagic fever, have emerged as a global public concern since the beginning of the century (Dantas-Torres et al. 2012; Dantas-Torres 2015). In parallel to progress made in the fields of diagnostic and therapeutic technologies (Bologheanu et al. 2020; Serretiello et al. 2020) and in vaccine design (De la Fuente et al. 2017), mathematical models and analyses have also been advanced, using differential equations to explore the long-term behaviors of tick-borne pathogen spread in the vector–host interaction (see Wu et al. 2013; Rosà and Pugliese 2007; Zhang and Wu 2020 and references therein).

Tick-borne diseases are mainly transmitted through the bite of ticks. Ticks, second only to mosquitoes for vectoring human, have two families: *Ixodidae* (hard ticks) and *Argasidae* (soft ticks) (Hoskins 1991). Approximately 650 *Ixodidae* species have been reported worldwide (Boulanger et al. 2019). Ticks in the family of *Ixodidae* distribute widely in natural ecosystems and take major responsibilities for transmitting a variety of tick-borne diseases. Most ticks go through four life stages: egg, larva, nymph and adult. The development in each post-egg stage involves a process of questing, attaching, feeding and engorging (Marquardt 2005), with female adult ticks laying thousands of eggs and dying shortly to complete the life cycle. Due to global warming, tick populations expand their spatial habitats and temporal activity season into winter months (Sagurova et al. 2019). For a better estimation of the tick-borne disease spread, understanding the tick population dynamics is in priority.

An important mechanism for potentially complex tick population dynamics is the developmental heterogeneity due to diapause, an important physiological process for ticks to respond to variable environmental conditions, such as changes in temperature, photoperiod, rainfall and host availability. Tick diapause was described for the first time by Beinarowitch (1907). There are two basic types of tick diapause: behavioral and developmental diapause, which delay host-questing following the transformation of molt and suspend development from one stage to the next, respectively (Dobson et al. 2001; Ogden et al. 2004). Korotkov (2015) estimated the duration of tick development cycle that can reach $3\sim 6$ years. Shu et al. (2020) proposed a delay differential equation for tick population dynamics with multiple delays and discussed global Hopf branches. Hoch et al. (2010) assumed that all engorged ticks experience diapause and develop until the second spring of next year and investigated the dynamics of tick population with temperature changes. Despite these efforts, quantifying impact of diapause on tick population dynamics remains a challenge partially because the diapause is linked to the experience of ticks in different habitats and environments during their entire life cycle.

Natural habitats have been increasingly separated into many smaller patches by human activities, such as road construction, mining, deforestation and aggressive agricultural cultivation. Although it is unlikely for ticks to move over a long distance, hosts carrying feeding ticks can freely move among different patches. Since the blood meal of hard ticks such as *Ixodes ricinus* lasts several days (Gray 1981), ticks can be carried over by their hosts and move from one patch to another during the period of blood feeding. Hence, it is highly possible that the patch where a tick quested and attached to a host is different from the patch where the tick drops after blood feeding and engorgement, and hence, the development delays are highly relevant to the patches and their ambient conditions. This calls for patch model formulation of the tick population dynamics.

Some earlier studies have proposed multi-patch population dynamics models, see Arino et al. (2019) and references therein. Guiver et al. (2017) gave an explanation of the effects of dispersal-induced coupling on population persistence across discrete patches. Wei and Wang (2020) described a single-species population model between two patches (the natural reserved and naturally environmental) and revealed the sensitivity of fluctuation intensity and migration rate on the population persistence and extinction. Smith et al. (2014) analyzed the long-term behavior of a structured two-patch metapopulation model and used the perturbation theory to discover an Allee effect for a two-patch model .

Here, taking into consideration of diapause development during some physiological stages of ticks in some specific patches the ticks stay in their life cycle, we propose a tick population dynamics patch model with delay. We incorporate both normal developmental delay and diapause delay in two different environments/patches between which ticks move because of the mobility of respective hosts. We show how tick populations are redistributed between questing and engorgement phases by the mobility patterns of the hosts, and we model the heterogeneity of tick development delays due to their distribution between these two patches, those with normal development and those with diapause delay. For such a coupled system of delay differential equations with multiple delays, we establish the existence and uniqueness of a positive equilibrium and obtain conditions for the local stability. We then conduct some numerical simulations to see how the host mobility and diapause development delay combined synchronize the coupled system to a synchronized mode of oscillation, and how this simplifies the pattern of oscillations in comparison with the situations when the two patches are not connected by host mobility.

## The Model

### Spatial Dynamics of Mobile Hosts

Consider a tick species for which the hosts of feeding ticks in different stages move between two geological patches, dispersing the engorged ticks among these patches characterized by differential development delays between consecutive stages.

For a given host population under consideration, we denote the number of respective host individuals in each of these two locations by $H^R(t)$ and $H^D(t)$ at time *t*, respectively, where we use sup-index *R* for patch facilitating regular development delay and sup-index *D* for patch facilitating diapause development delay of the ticks in a given developmental stage (larval, nymphal and adult). Ignoring all other population dynamics, the spatial dynamics of the hosts is given by

$$\begin{aligned} \begin{aligned} \dfrac{dH^R(t)}{dt}=m_{h2}H^D(t)-m_{h1}H^R(t),\\ \dfrac{dH^D(t)}{dt}=m_{h1}H^R(t)-m_{h2}H^D(t), \end{aligned} \end{aligned}$$

with $m_{h1}$ and $m_{h2}$ for the mobility rates of the hosts from patch R to patch D, and from patch D to patch R, respectively. It follows that

$$\begin{aligned} \begin{pmatrix}H^R(t) \\ H^D(t) \end{pmatrix}=e^{T_{m_h}(t-s)}\begin{pmatrix}H^R(s) \\ H^D(s) \end{pmatrix}, \qquad T_{m_h}=\begin{pmatrix}-m_{h1} &{} m_{h2} \\ m_{h1} &{} -m_{h2} \end{pmatrix}. \end{aligned}$$

Based on the method for calculating basic solution matrix of linear ordinary differential equations (Sina and Ali 2003), we have

$$\begin{aligned} e^{T_{m_h}(t-s)}=\dfrac{1}{m_{h1}+m_{h2}}\Big [\begin{pmatrix}m_{h2} &{} m_{h2}\\ m_{h1} &{} m_{h1}\end{pmatrix}+e^{-(m_{h1}+m_{h2})(t-s)}\begin{pmatrix}m_{h1} &{} -m_{h2}\\ -m_{h1} &{} m_{h2}\end{pmatrix}\Big ], \end{aligned}$$

which describes the redistribution dynamics of host individuals (and hence the respective engorged ticks) as follows:

$$\begin{aligned} \begin{aligned} H^R(t)&=\dfrac{1}{m_{h1}+m_{h2}}[m_{h2}(H^R(s)+H^D(s))+e^{-(m_{h1}+m_{h2})(t-s)}(m_{h1}H^R(s)\\&\quad -m_{h2}H^D(s))],\\ H^D(t)&=\dfrac{1}{m_{h1}+m_{h2}}[m_{h1}(H^R(s)+H^D(s))-e^{-(m_{h1}+m_{h2})(t-s)}(m_{h1}H^R(s)\\&\quad -m_{h2}H^D(s))]. \end{aligned} \end{aligned}$$

### Tick Population Dynamics with Feeding Tick’s Mobility Driven by Hosts

**Fig. 1:**
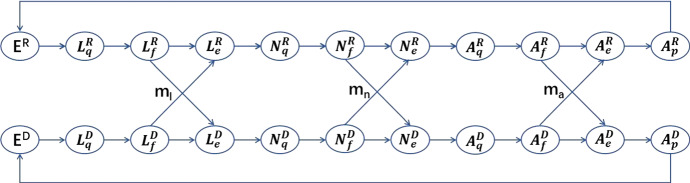
A schematic illustration of tick population dynamics with mobility driven by host migration between patch R and patch D: female producing adult ticks after breeding on a host drop to the ground to lay eggs, and then, eggs hatch into the larval ticks. Larvae ($L_q$) start to quest their first host for an initial blood meal. After feeding, larval ticks ($L_f$) become engorged larval ticks ($L_e$) and molt into nymphs. Nymphs experience the same procedure, questing ($N_q$), feeding ($N_f$) and engorged ($N_e$), for a second blood meal, and eventually molt into adult ticks. The adult ticks ($A_q$) then hunt a larger host, such as deer, where they are able to feed ($A_f$) and become engorged ($A_e$), and finally drop from host for the reproduction ($A_p$). There are three mobility patterns, characterized by the random movements of host for feeding larvae, nymphs and adult ticks with migration rates $m_l$, $m_n$ and $m_a$, respectively

We now consider a tick species in a region with two distinct patches where the environmental conditions are suitable for a regular development delay (normally one year) in patch R and a diapause delay (normally two years) in patch D for ticks in each development stage from larva to nymph, and from nymph to adult. We ignore the mobility of ticks during their engorged and questing activity stages, but ticks feeding on hosts move along the hosts which we assume move between the two patches with appropriate mobility rates $m_l$, $m_n$ and $m_a$ that will further be stratified in terms of the movement from patch R to D or from patch D to R. The flowchart of the evolution of tick population between the two patches with mobility driven by the host migration is given in Fig. 1.

Let $\tau _{el}^R$ and $\tau _{el}^D$ ($\tau _{el}^R<\tau _{el}^D$) be the durations which eggs hatch into questing larval ticks and then become feeding larval ticks for the first blood meal on the patch R and patch D, respectively. $\rho _{efl}^R$ and $\rho _{efl}^D$ are corresponding probabilities of survivability from eggs to feeding larval ticks in the patch R and patch D, respectively. The dynamics of feeding larval ticks takes the following form

1 $$\begin{aligned} \begin{pmatrix} L_f^R(t) \\ L_f^D(t) \end{pmatrix} =\begin{pmatrix} \rho _{efl}^R &{} 0 \\ 0 &{} \rho _{efl}^D \end{pmatrix}\begin{pmatrix} E^R(t-\tau _{el}^R) \\ E^D(t-\tau _{el}^D) \end{pmatrix}. \end{aligned}$$

Since blood feeding usually takes several days, larval ticks are able to be carried over by their hosts during this period. Let $\tau _{fl}$ be the feeding time of larval ticks and $m_l$ be the migration probability of host population between the two patches, then we have

$$\begin{aligned} \begin{pmatrix} L_e^R(t) \\ L_e^D(t) \end{pmatrix} =e^{T_{m_l}\cdot \tau _{fl}}\begin{pmatrix} L_f^R(t-\tau _{fl}) \\ L_f^D(t-\tau _{fl}) \end{pmatrix}, \quad T_{m_l}=\begin{pmatrix}-m_{l1} &{} m_{l2} \\ m_{l1} &{} -m_{l2} \end{pmatrix}. \end{aligned}$$

After the engorged larval ticks fall to the ground, they develop into nymphs with the survival probabilities $\rho _{efn}^R$ and $\rho _{efn}^D$, respectively. The corresponding developmental delays are $\tau _{en}^R$ and $\tau _{en}^D$ ($\tau _{en}^R<\tau _{en}^D$) on each patch. The evolution can be described as follows

$$\begin{aligned} \begin{pmatrix} N_f^R(t) \\ N_f^D(t) \end{pmatrix} =\begin{pmatrix} \rho _{efn}^R &{} 0 \\ 0 &{} \rho _{efn}^D \end{pmatrix}\begin{pmatrix} L_e^R(t-\tau _{en}^R) \\ L_e^D(t-\tau _{en}^D) \end{pmatrix}. \end{aligned}$$

Similarly, let $m_n$ and $m_a$ be the migration probabilities of host population which can carry feeding nymphal ticks and feeding adult ticks and redistribute them in patch R and patch D when feeding ticks become engorged and drop off from the hosts and $\tau _{fn}$ and $\tau _{fa}$ be the feeding durations of nymphal ticks and adult ticks, respectively. Let $\rho _{efa}^R$ and $\rho _{efa}^D$ be survival probabilities of adult ticks developing from engorged nymphs and $\tau _{ea}^{R}$ and $\tau _{ea}^{D}$ be the corresponding development time delays. Then, we yield

$$\begin{aligned}&\begin{pmatrix} N_e^R(t) \\ N_e^D(t) \end{pmatrix} =e^{T_{m_n}\cdot \tau _{fn}}\begin{pmatrix} N_f^R(t-\tau _{fn}) \\ N_f^D(t-\tau _{fn}) \end{pmatrix},\\&\begin{pmatrix} A_f^R(t) \\ A_f^D(t) \end{pmatrix} =\begin{pmatrix} \rho _{efa}^R &{} 0 \\ 0 &{} \rho _{efa}^D \end{pmatrix}\begin{pmatrix} N_e^R(t-\tau _{ea}^R) \\ N_e^D(t-\tau _{ea}^D) \end{pmatrix}, \end{aligned}$$

and

$$\begin{aligned} \begin{pmatrix} A_e^R(t) \\ A_e^D(t) \end{pmatrix} =e^{T_{m_a}\cdot \tau _{fa}}\begin{pmatrix} A_f^R(t-\tau _{fa}) \\ A_f^D(t-\tau _{fa}) \end{pmatrix}, \end{aligned}$$

where $$T_{m_n}=\begin{pmatrix}-m_{n1} &{} m_{n2} \\ m_{n1} &{} -m_{n2} \end{pmatrix}$$ and $$T_{m_a}=\begin{pmatrix}-m_{a1} &{} m_{a2} \\ m_{a1} &{} -m_{a2} \end{pmatrix}$$.

Following engorgement, adult ticks leave from host and start to be ready for their reproduction. Here, let $\rho _{epa}^R$ and $\rho _{epa}^D$ be development rates from engorged adults to egg-producing adults, and the corresponding development time delays are $\tau _{epa}^R$ and $\tau _{epa}^D$, respectively. We describe this process by

2 $$\begin{aligned} \begin{pmatrix} A_p^R(t) \\ A_p^D(t) \end{pmatrix} =\begin{pmatrix} \rho _{epa}^R &{} 0 \\ 0 &{} \rho _{epa}^D \end{pmatrix}\begin{pmatrix} A_e^R(t-\tau _{epa}^R) \\ A_e^D(t-\tau _{epa}^D) \end{pmatrix}. \end{aligned}$$

Finally, these egg-producing adult ticks will reproduce by laying eggs and die shortly after that. The dynamics of eggs can be written as

3 $$\begin{aligned} \dfrac{d}{dt}\begin{pmatrix}E^R(t)\\ E^D(t)\end{pmatrix} =\begin{pmatrix}f^R(A_p^R(t))\\ f^D(A_p^D(t))\end{pmatrix}-\begin{pmatrix}\mu ^RE^R(t)\\ \mu ^DE^D(t)\end{pmatrix}, \end{aligned}$$

where $f^R$ and $f^D$ are reproduction functions on patch R and patch D and both use with Ricker functions, that is,

$$\begin{aligned} \begin{aligned} f^R(x)=p^Rxe^{-s^Rx}, ~~~ x\ge 0,\\ f^D(x)=p^Dxe^{-s^Dx}, ~~~ x\ge 0, \end{aligned} \end{aligned}$$

where $p^R$ and $p^D$ are maximal numbers of eggs that an egg-producing female adult tick can lay per unit time, $s^R$ and $s^D$ represent the strengths of density dependence, and $\mu ^R$ and $\mu ^D$ are exit rates of eggs including mortality rate and developmental rate from egg to larval stage.

From Eqs. (1)–(2), we have

$$\begin{aligned} \begin{pmatrix} A_p^R(t)\\ A_p^D(t) \end{pmatrix}=Q \begin{pmatrix} E^R(t-\tau ^R)\\ E^D(t-\tau ^D) \end{pmatrix}, \end{aligned}$$

where $$Q=\begin{pmatrix} q_{11} &{} q_{12}\\ q_{21} &{} q_{22} \end{pmatrix} =\begin{pmatrix} \rho _{epa}^R &{} 0\\ 0 &{} \rho _{epa}^D \end{pmatrix} \cdot e^{T_{m_a}\tau _{f_a}} \cdot \begin{pmatrix} \rho _{efa}^R &{} 0\\ 0 &{} \rho _{efa}^D \end{pmatrix} \cdot e^{T_{m_n}\tau _{f_n}} \cdot \begin{pmatrix} \rho _{efn}^R &{} 0\\ 0 &{} \rho _{efn}^D \end{pmatrix} \cdot e^{T_{m_l}\tau _{f_l}} \cdot \begin{pmatrix} \rho _{efl}^R &{} 0\\ 0 &{} \rho _{efl}^D \end{pmatrix},$$

$$\tau ^R=\tau _{epa}^R+\tau _{fa}+\tau ^R_{ea}+\tau _{fn}+\tau ^R_{en}+\tau _{fl}+\tau _{el}^R$$,

$$\tau ^D=\tau _{epa}^D+\tau _{fa}+\tau ^D_{ea}+\tau _{fn}+\tau ^D_{en}+\tau _{fl}+\tau _{el}^D$$,

and then, system (3) can be rewritten in following form

4 $$\begin{aligned} \begin{pmatrix}{\dot{E}}^R(t)\\ {\dot{E}}^D(t)\end{pmatrix} =\begin{pmatrix}f^R(q_{11}E^R(t-\tau ^R)+q_{12}E^D(t-\tau ^D))-\mu ^RE^R(t)\\ f^D(q_{21}E^R(t-\tau ^R)+q_{22}E^D(t-\tau ^D))-\mu ^DE^D(t) \end{pmatrix}. \end{aligned}$$

## The Case of Distinct Mobility of Hosts for Feeding Adult Ticks

An important case is when regular development takes place in patch R, while diapause development occurs in patch D. Let the corresponding time delays be $\tau ^R$ and $\tau ^D$ for the entire life cycle, respectively. This naturally leads to our standing assumption in this study: $\tau ^D=2\tau ^R$. Using $\tau =\tau ^R$, we have $\tau ^D=2\tau$. Since the survival probability in patch D is smaller comparing with that in patch R, we write

5 $$\begin{aligned} \begin{aligned} \rho _{efl}^D=\kappa _{l}\rho _{efl}^R, ~~ \rho _{efn}^D=\kappa _{n}\rho _{efn}^R, ~~ \rho _{efa}^D=\kappa _{a}\rho _{efa}^R, ~~ \rho _{epa}^D=\kappa _{p}\rho _{epa}^R,\\ \end{aligned} \end{aligned}$$

with $\kappa _l, \kappa _n, \kappa _a, \kappa _p\in (0,1)$. We assume two patches do not have any distinction for the birth characteristics (the maximum reproduction numbers of eggs, the exit rates from the egg stage and the strengths of density dependence), leading to

6 $$\begin{aligned} \mu ^R=\mu ^D, ~~ p^R=p^D, ~~ s^R=s^D. \end{aligned}$$

We do not focus on the case of insignificant mobilities of hosts for larval and nymphal ticks, but assume there is a preference of hosts for feeding adult ticks moving from patch R to patch D. Therefore, we have

$$\begin{aligned} \begin{aligned} m_{l1}&=m_{l2}=0, \quad m_{n1}=m_{n2}=0,\\ m_{a1}&={\bar{m}}_a(1+\delta ), \quad m_{a2}={\bar{m}}_a(1-\delta ), \end{aligned} \end{aligned}$$

where ${{\bar{m}}}_a$ is the average mobility rate of the hosts for feeding adult ticks between the two patches and $0<\delta <1$. We can now drop all the subscripts of the parameters in (5) and (6), and we can also calculate all entries in matrix *Q* as follows:

$$\begin{aligned} \begin{aligned} q_{11}&=\rho \left( \dfrac{1}{2}-\dfrac{\delta }{2}+\dfrac{1+\delta }{2}e^{-\varDelta }\right) ,\\ q_{12}&=k_{a}k_nk_l\rho \left( \dfrac{1}{2}-\dfrac{\delta }{2}-\dfrac{1-\delta }{2}e^{-\varDelta }\right) ,\\ q_{21}&=k_{p}\rho \left( \dfrac{1}{2}+\dfrac{\delta }{2}-\dfrac{1+\delta }{2}e^{-\varDelta }\right) ,\\ q_{22}&=k_pk_{a}k_nk_l\rho \left( \dfrac{1}{2}+\dfrac{\delta }{2}+\dfrac{1-\delta }{2}e^{-\varDelta }\right) , \end{aligned} \end{aligned}$$

where

$$\begin{aligned} \rho =\rho _{epa}^R\rho _{efa}^R\rho _{efn}^R\rho _{efl}^R,\quad \varDelta =2{{\bar{m}}}_{a}\tau _{fa}. \end{aligned}$$

### Equilibrium Analyses

The equilibria of model (4) are determined by the following nonlinear equations

7 $$\begin{aligned} \begin{aligned} p(q_{11}E^R+q_{12}E^D)e^{-s(q_{11}E^R+q_{12}E^D)}=\mu E^R,\\ p(q_{21}E^R+q_{22}E^D)e^{-s(q_{21}E^R+q_{22}E^D)}=\mu E^D. \end{aligned} \end{aligned}$$

Clearly, model (4) always has a trivial equilibrium (0, 0).

Let

8 $$\begin{aligned} \begin{aligned} y_1=q_{11}E^R+q_{12}E^D,\\ y_2=q_{21}E^R+q_{22}E^D, \end{aligned} \end{aligned}$$

namely,

9 $$\begin{aligned} \begin{aligned} E^R=u_{11}y_1+u_{12}y_2,\\ E^D=u_{21}y_1+u_{22}y_2, \end{aligned} \end{aligned}$$

where

$$\begin{aligned} \begin{aligned} u_{11}=\dfrac{1+\delta +(1-\delta )e^{-\varDelta }}{2\rho e^{-\varDelta }}, \\ u_{12}=-\dfrac{(1-\delta )(1-e^{-\varDelta })}{2k_p\rho e^{-\varDelta }} ,\\ u_{21}=-\dfrac{(1+\delta )(1-e^{-\varDelta })}{2k_ak_nk_l\rho e^{-\varDelta }},\\ u_{22}=\dfrac{1-\delta +(1+\delta )e^{-\varDelta }}{2k_pk_ak_nk_l\rho e^{-\varDelta }}. \end{aligned} \end{aligned}$$

Substituting (8) into equilibrium equation (7), we have

10 $$\begin{aligned} \begin{aligned} py_1e^{-sy_1}=\mu (u_{11}y_1+u_{12}y_2),\\ py_2e^{-sy_2}=\mu (u_{21}y_1+u_{22}y_2), \end{aligned} \end{aligned}$$

namely

11 $$\begin{aligned} \begin{aligned} y_1=u_{21}^{-1}(p\mu ^{-1}y_2e^{-sy_2}-u_{22}y_2)=J(y_2),\\ y_2=u_{12}^{-1}(p\mu ^{-1}y_1e^{-sy_1}-u_{11}y_1)=R(y_1). \end{aligned} \end{aligned}$$

**Fig. 2:**
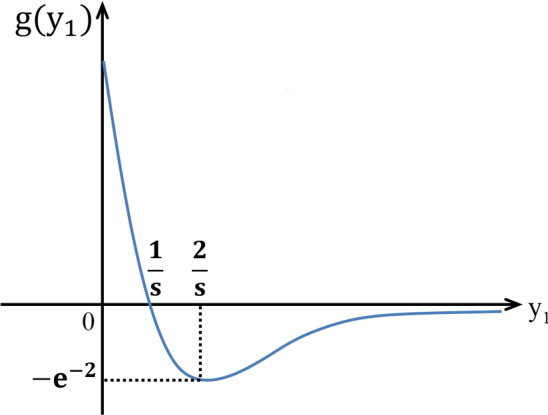
A schematic illustration of the function $g(y_1)$

From the expression of the function $R(y_1)$, we can get

$$\begin{aligned} R'(y_1)=\mu ^{-1}u_{12}^{-1}p\left[ g(y_1)-\dfrac{\mu u_{11}}{p}\right] , \end{aligned}$$

and

$$\begin{aligned} R''(y_1)=-\mu ^{-1}u_{12}^{-1}pse^{-sy_1}[2-sy_1], \end{aligned}$$

where $g(y_1)=e^{-sy_1}(1-sy_1)$. Figure 2 gives a schematic diagram of the function $g(y_1)$. The maximum value of $g(y_1)$ is 1 at $y_1=0$ and minimum $-e^{-2}$ at $y_1=2/s$. $g(y_1)$ is a decreasing function of $y_1\in [0,2/s]$ and then increasing in the interval $(2/s,+\infty )$ with the limiting value $\displaystyle \lim _{y_1\rightarrow +\infty }g(y_1)=0$.

**Fig. 3:**
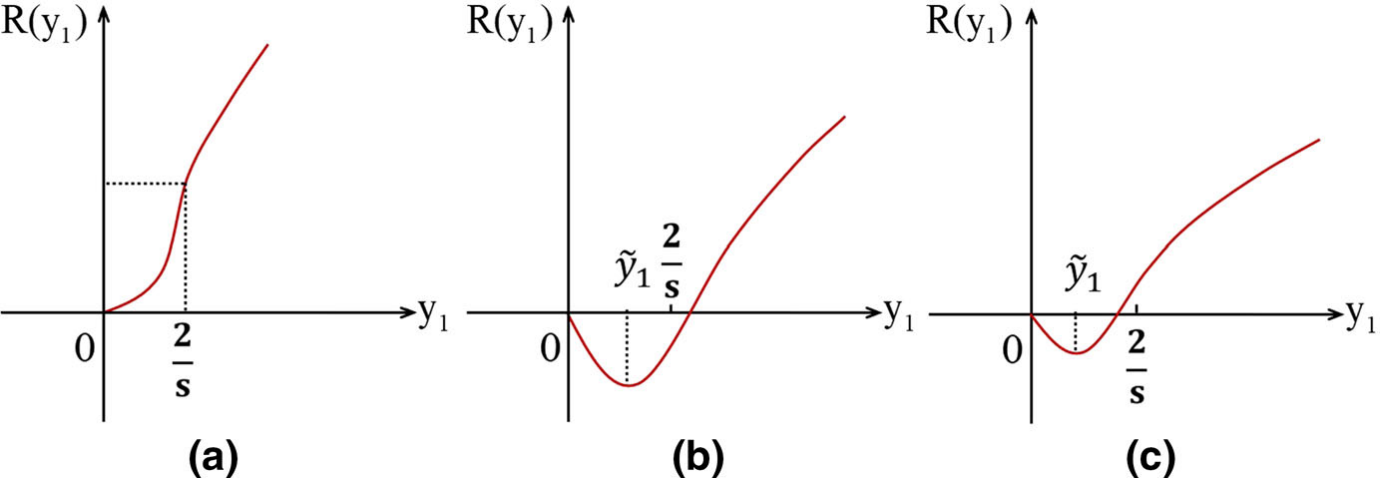
A schematic illustration of the function $R(y_1)$

Thus, we obtain the following important properties of $R(y_1)$:

(i) if $\dfrac{\mu u_{11}}{p}>1$, then $g(y_1)-\dfrac{\mu u_{11}}{p}<0$. From the expression of (9), it is clear that $u_{12}<0$, so $R'(y_1)>0$ for any $y_1>0$, i.e., $R(y_1)$ is a monotone increasing function in $(0,+\infty )$. Note that $R(0)=0$, $\displaystyle \lim _{y_1\rightarrow +\infty }R'(y_1)=-u_{12}^{-1}u_{11}$ and $\dfrac{s}{2}$ is an inflection point. The description of $R(y_1)$ is displayed in Fig. 3a.
(ii) if $0<\dfrac{\mu u_{11}}{p}\le 1$, there exists only one zero solution ${\tilde{y}}_{1}(<1/s)$ such that $R'({\tilde{y}}_{1})=0$. Clearly, $R(0)=0$ and $\displaystyle \lim _{y_1\rightarrow +\infty }R'(y_1)=-u_{12}^{-1}u_{11}$.

Table 1 describes properties of the curves of $R(y_1)$, $R'(y_1)$ and $R''(y_1)$. Accordingly, we have:

- $\blacktriangleright$ if $0<\dfrac{\mu u_{11}}{p}< e^{-2}$, then $R(\dfrac{2}{s})<0$ (see Fig. 3b);
- $\blacktriangleright$ if $e^{-2}\le \dfrac{\mu u_{11}}{p}\le 1$, then $R(\dfrac{2}{s})\ge 0$ (see Fig. 3c).

**Table 1:** Properties of $R(y_1)$, $R'(y_1)$ and $R''(y_1)$

$y_1$	$(0,{\tilde{y}}_{1})$	${\tilde{y}}_{1}$	$({\tilde{y}}_{1},\dfrac{2}{s})$	$\dfrac{2}{s}$	$(\dfrac{2}{s}, +\infty )$
$R(y_1)$	decreasing&	minimum	increasing&	inflection	increasing&
	concave upward	value	concave upward	point	concave downward
$R'(y_1)$	−	0	$+$	$+$	$+$
$R''(y_1)$	$+$	$+$	$+$	0	−

Similarly, for $J(y_2)$, we get

$$\begin{aligned} \begin{aligned} J'(y_2)&=\mu ^{-1}u_{21}^{-1}p\left[ g(y_2)-\dfrac{\mu u_{22}}{p}\right] ,\\ J''(y_2)&=-\mu ^{-1}u_{21}^{-1}pse^{-sy_2}[2-sy_2]. \end{aligned} \end{aligned}$$

Therefore,

$$\begin{aligned} J'(+\infty )=-u_{21}^{-1}u_{22}=\dfrac{1-\delta +(1+\delta )e^{-\varDelta }}{k_p(1+\delta )(1-e^{-\varDelta })}. \end{aligned}$$

Comparing with

$$\begin{aligned} R'(+\infty )=-u_{12}^{-1}u_{11}=k_p\dfrac{1+\delta +(1-\delta )e^{-\varDelta }}{(1-\delta )(1-e^{-\varDelta })}, \end{aligned}$$

it follows

$$\begin{aligned} R'(+\infty )J'(+\infty )>1. \end{aligned}$$

**Fig. 4:**
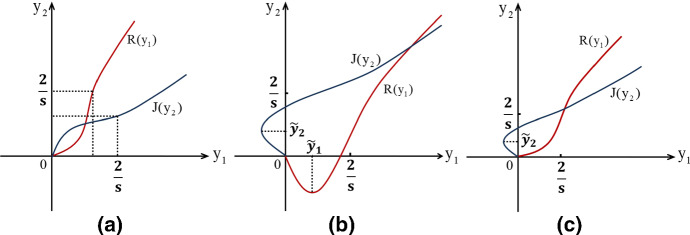
The scenarios of relative locations and interactions of the functions $R(y_1)$ and $J(y_2)$

Thus, there exists a unique positive equilibrium $(y_1^*, y_2^*)$ for (10). Based on the analysis for $J(y_2)$ and $R(y_1)$, we have the following conclusions about the location of the unique positive equilibrium $(y_1^*, y_2^*)$:

(iii) if $\min \{\dfrac{\mu u_{11}}{p},\dfrac{\mu u_{22}}{p}\}>1$, Fig. 4a plots the graphs of $J(y_2)$ and $R(y_1)$. if $0<\max \{\dfrac{\mu u_{11}}{p},\dfrac{\mu u_{22}}{p}\}\le 1$, Fig. 4b describes the schematic diagram of $J(y_2)$ and $R(y_1)$.
(iv) if $0<\min \{\dfrac{\mu u_{11}}{p},\dfrac{\mu u_{22}}{p}\}< 1<\max \{\dfrac{\mu u_{11}}{p},\dfrac{\mu u_{22}}{p}\}$, without loss of generality, we may assume that $u_{22}<u_{11}$. Figure 4c describes the schematic diagram of $J(y_2)$ and $R(y_1)$.

In summary, we have the following result:

#### Theorem 1

There exists a unique positive equilibrium $(E_*^R, E_*^D)$ for model (4).

### Stability

The linearized system (4) at the tick-free equilibrium $P_0=(0,0)$ is given by

12 $$\begin{aligned} \begin{pmatrix}{\dot{E}}^R(t)\\ {\dot{E}}^D(t)\end{pmatrix} =\begin{pmatrix}pq_{11}E^R(t-\tau )+pq_{12}E^D(t-2\tau )-\mu E^R(t)\\ pq_{21}E^R(t-\tau )+pq_{22}E^D(t-2\tau )-\mu E^D(t) \end{pmatrix}. \end{aligned}$$

This gives a linear system of delay differential equations with positive feedback as the coefficient matrix

$$\begin{aligned} p\begin{pmatrix}q_{11} &{} q_{12}\\ q_{21} &{} q_{22}\end{pmatrix}>0. \end{aligned}$$

Therefore, the stability of the zero solution of (12) is equivalent to the stability of the zero solution for the corresponding linear ordinary differential equation

13 $$\begin{aligned} \begin{pmatrix}{\dot{E}}^R(t)\\ {\dot{E}}^D(t)\end{pmatrix} =\begin{pmatrix}pq_{11}E^R(t)+pq_{12}E^D(t)-\mu E^R(t)\\ pq_{21}E^R(t)+pq_{22}E^D(t)-\mu E^D(t) \end{pmatrix}. \end{aligned}$$

The characteristic equation of model (13) at the zero equilibrium is

$$\begin{aligned} (\lambda +\mu )^2-p\rho \zeta (\lambda +\mu )+k_pk_ak_nk_lp^2\rho ^2e^{-\varDelta }=0, \end{aligned}$$

where

$$\begin{aligned} \zeta =\dfrac{1}{2}[(1-\delta +(1+\delta )e^{-\varDelta })+k_pk_ak_nk_l(1+\delta +(1-\delta )e^{-\varDelta })]. \end{aligned}$$

Moreover, we have

$$\begin{aligned} \begin{aligned}&\zeta ^2-4k_pk_ak_nk_le^{-\varDelta }\\&\quad =\dfrac{1}{4}[(1-\delta +(1+\delta )e^{-\varDelta })^2+k_p^2k_a^2k_n^2k_l^2(1+\delta +(1-\delta )e^{-\varDelta })^2] \\&\qquad +2k_pk_ak_nk_l\left( \dfrac{1-\delta ^2}{4}+\dfrac{1+\delta ^2}{2}e^{-\varDelta }+\dfrac{1-\delta ^2}{4}e^{-2\varDelta }\right) \\&\qquad -4k_pk_ak_nk_le^{-\varDelta }\\&\quad \ge \dfrac{1}{4}[(1-\delta +(1+\delta )e^{-\varDelta })-k_pk_ak_nk_l(1+\delta +(1-\delta )e^{-\varDelta })]^2\ge 0. \end{aligned} \end{aligned}$$

Then, the two eigenvalues of (13) are

$$\begin{aligned} \lambda =-\mu +\dfrac{1}{2}p\rho \zeta \pm \dfrac{1}{2}p\rho \sqrt{\zeta ^2-4k_pk_ak_nk_le^{-\varDelta }}. \end{aligned}$$

Clearly, the two eigenvalues are both negative if the following condition is satisfied

14 $$\begin{aligned} \mu ^2-p\rho \zeta \mu +p^2\rho ^2k_pk_ak_nk_le^{-\varDelta }>0. \end{aligned}$$

Thus, we have established the following result:

#### Theorem 2

Assume (14) holds. Then the trivial equilibrium (0, 0) of model (4) is locally asymptotically stable.

In what follows, we consider the stability of the positive equilibrium $P_*$ of model (4). Translating the positive equilibrium into the origin through introducing $x_1(t)=E^R(t)-E^R_*$ and $x_2(t)=E^D(t)-E^D_*$ in model (4) and linearizing it, we have

15 $$\begin{aligned} \begin{pmatrix}{\dot{x}}_1(t)\\ {\dot{x}}_2(t)\end{pmatrix} =\begin{pmatrix}\gamma _1q_{11} &{} \gamma _1q_{12}\\ \gamma _2q_{21} &{} \gamma _2q_{22}\end{pmatrix}\begin{pmatrix}x_1(t-\tau )\\ x_2(t-2\tau )\end{pmatrix}-\mu \begin{pmatrix}x_1(t)\\ x_2(t)\end{pmatrix}, \end{aligned}$$

where

$$\begin{aligned} \gamma _1=pe^{-s(q_{11}E_*^R+q_{12}E_*^D)}-s\mu E_*^R, \;\; \gamma _2=pe^{-s(q_{21}E_*^R+q_{22}E_*^D)}-s\mu E_*^D. \end{aligned}$$

We can further compute

$$\begin{aligned} \begin{aligned} \gamma _1&=\dfrac{\mu E_*^R}{q_{11}E_*^R+q_{12}E_*^D}-s\mu E_*^R=\left( \dfrac{1}{q_{11}E_*^R+q_{12}E_*^D}-s\right) \mu E_*^R,\\ \gamma _2&=\dfrac{\mu E_*^R}{q_{21}E_*^R+q_{22}E_*^D}-s\mu E_*^D=\left( \dfrac{1}{q_{21}E_*^R+q_{22}E_*^D}-s\right) \mu E_*^D. \end{aligned} \end{aligned}$$

It is clear that (15) is a positive feedback system if the following condition is satisfied

16 $$\begin{aligned} \left\{ \begin{array}{ll} q_{11}E_*^R+q_{12}E_*^D<\dfrac{1}{s},\\ q_{21}E_*^R+q_{22}E_*^D<\dfrac{1}{s}. \end{array} \right. \end{aligned}$$

From (8) and (11), the condition (16) can be guaranteed if

$$\begin{aligned} J(s^{-1})>s^{-1},\quad R(s^{-1})>s^{-1}, \end{aligned}$$

which are equivalent to

17 $$\begin{aligned} \left\{ \begin{array}{ll} \dfrac{p}{\mu e}<\dfrac{k_p((1+\delta )e^\varDelta +1-\delta )-(1-\delta )(e^{\varDelta }-1)}{2k_p\rho },\\ \dfrac{p}{\mu e}<\dfrac{(1-\delta )e^{\varDelta } +(1+\delta )-k_p(1+\delta )(e^{\varDelta }-1)}{2k_pk_ak_nk_l\rho }. \end{array} \right. \end{aligned}$$

Then, the stability of the positive equilibrium $(E_*^R, E_*^D)$ can be determined by the stability of the zero solution to the following linear system of ODE equations:

18 $$\begin{aligned} \begin{pmatrix}{\dot{x}}_1(t)\\ {\dot{x}}_2(t)\end{pmatrix} =\begin{pmatrix}\gamma _1q_{11} &{} \gamma _1q_{12}\\ \gamma _2q_{21} &{} \gamma _2q_{22}\end{pmatrix}\begin{pmatrix}x_1(t)\\ x_2(t)\end{pmatrix}-\mu \begin{pmatrix}x_1(t)\\ x_2(t)\end{pmatrix}. \end{aligned}$$

The characteristic equation of (18) is

$$\begin{aligned} (\lambda -\gamma _1q_{11}+\mu )(\lambda -\gamma _2q_{22}+\mu )-\gamma _1\gamma _2q_{12}q_{21}=0. \end{aligned}$$

Its roots are

$$\begin{aligned} \lambda _{1,2}=-\mu +\dfrac{\gamma _1q_{11}+\gamma _2q_{22}}{2}\pm \sqrt{\dfrac{(\gamma _1q_{11} -\gamma _2q_{22})^2}{4}+\gamma _1\gamma _2q_{12}q_{21}}. \end{aligned}$$

Since (17) implies $\gamma _1$ and $\gamma _2$ are both positive, the stability of the positive equilibrium $(E_*^R,E_*^D)$ depends on whether

19 $$\begin{aligned} -\mu +\dfrac{\gamma _1q_{11}+\gamma _2q_{22}}{2}+\sqrt{\dfrac{(\gamma _1q_{11}-\gamma _2q_{22})^2}{4}+\gamma _1\gamma _2q_{12}q_{21}}<0. \end{aligned}$$

(19) can be rewritten as

$$\begin{aligned} \mu ^2+\gamma _1\gamma _2(q_{11}q_{22}-q_{12}q_{21})-\mu (\gamma _1q_{11}+\gamma _2q_{22})>0, \end{aligned}$$

which is equivalent to

20 $$\begin{aligned} \begin{aligned}&1-\rho \left[ \left( \dfrac{1}{q_{11}E_*^R+q_{12}E_*^D}-s\right) E_*^R\left( \dfrac{1-\delta }{2}+\dfrac{1+\delta }{2}e^{-\varDelta }\right) \right. \\&\quad \left. +\left( \dfrac{1}{q_{21}E_*^R+q_{22}E_*^D}-s\right) k_pk_ak_nk_lE_*^D\left( \dfrac{1+\delta }{2}+\dfrac{1-\delta }{2}e^{-\varDelta }\right) \right] \\&\quad +\left( \dfrac{1}{q_{11}E_*^R+q_{12}E_*^D}-s\right) \left( \dfrac{1}{q_{21}E_*^R+q_{22}E_*^D}-s\right) k_pk_ak_nk_lE_*^RE_*^D\rho ^2e^{-\varDelta }>0. \end{aligned} \end{aligned}$$

It is easy to see that (20) is satisfied if the following condition holds

21 $$\begin{aligned} 1-\rho [(q_{11}E_*^R+q_{12}E_*^D-s)E_*^R+(q_{21}E_*^R+q_{22}E_*^D-s)k_pk_ak_nk_lE_*^D]>0. \end{aligned}$$

Therefore, we have

#### Theorem 3

Assume that (17) and (21) hold. Then, the positive equilibrium $P_*(E_*^R,E_*^D)$ is locally stable.

We now consider the case when the condition of a positive feedback system is not satisfied. The characterization equation of (15) is

22 $$\begin{aligned} (\lambda +\mu -\gamma _1q_{11}e^{-\lambda \tau })(\lambda +\mu -\gamma _2q_{22}e^{-2\lambda \tau }) -\gamma _1\gamma _2q_{12}q_{21}e^{-3\lambda \tau }=0. \end{aligned}$$

From the analysis above, we can see that when $\tau =0$, the positive equilibrium $P_*$ is locally asymptotically stable if (21) is satisfied. In the absence of a positive feedback at the non-trivial equilibrium, Hopf bifurcation can take place when the characteristic equation has a pair of purely imaginary zeros. We now discuss whether (22) has a pair of purely imaginary roots.

Assume that a purely imaginary solution with the form $\lambda =i\omega$ where $\omega >0$ exists in Eq. (22). Substituting the purely imaginary solution into Eq. (22) and separating the real and imaginary parts, we can obtain

23 $$\begin{aligned} \left\{ \begin{aligned}&-\omega ^2+\mu ^2-[\mu (\gamma _1q_{11}\cos \omega \tau +\gamma _2q_{22}\cos 2\omega \tau )+\omega (\gamma _1q_{11}\sin \omega \tau +\gamma _2q_{22}\sin 2\omega \tau )]\\&\quad +\gamma _1\gamma _2(q_{11}q_{22}-q_{12}q_{21})\cos 3\omega \tau =0,\\&2\omega \mu -[\omega (\gamma _1q_{11}\cos \omega \tau +\gamma _2q_{22}\cos 2\omega \tau ) -\mu (\gamma _1q_{11}\sin \omega \tau +\gamma _2q_{22}\sin 2\omega \tau )]\\&\quad -\gamma _1\gamma _2(q_{11}q_{22}-q_{12}q_{21})\sin 3\omega \tau =0. \end{aligned} \right. \end{aligned}$$

Define

$$\begin{aligned} \begin{aligned} f(\omega )&=\mu (\gamma _1q_{11}\cos \omega \tau +\gamma _2q_{22}\cos 2\omega \tau )+\omega (\gamma _1q_{11} \sin \omega \tau +\gamma _2q_{22}\sin 2\omega \tau ),\\ g(\omega )&=\omega (\gamma _1q_{11}\cos \omega \tau +\gamma _2q_{22}\cos 2\omega \tau )-\mu (\gamma _1q_{11} \sin \omega \tau +\gamma _2q_{22}\sin 2\omega \tau ), \end{aligned} \end{aligned}$$

then Eq. (23) can be rewritten into the following form

24 $$\begin{aligned} \left\{ \begin{aligned}&-\omega ^2+\mu ^2-f(\omega )=-\gamma _1\gamma _2(q_{11}q_{22}-q_{12}q_{21})\cos 3\omega \tau ,\\&2\omega \mu -g(\omega )=\gamma _1\gamma _2(q_{11}^2-q_{12}^2)\sin 3\omega \tau . \end{aligned} \right. \end{aligned}$$

Since $\gamma _1$ and $\gamma _2$ are both functions of $E_*^R$ and $E_*^D$, it is difficult to solve the transcendental equation (24) in closed forms. However, given our proof of the unique positive equilibrium, all relevant coefficients are specifically given once model parameters are given. Therefore, we can develop a continuation procedure to locate the minimal value of $\tau$, for a given mobility $\delta \in [0,1]$, where (24) has a solution $\omega >0$. In particular, since ${\bar{m}}_a=0$ corresponds to the situation when two patches are isolated from each other, there exists a critical value $\tau ^R_*$ of delay $\tau$ such that the population starts to oscillate in patch R if $\tau >\tau ^R_*$, and there exists a critical value $\tau ^D_*$ of the delay such that the population starts to oscillate in patch D if $\tau >\tau ^D_*$ in patch D and if $\tau >\tau ^*_D$ (see Zhang and Wu (2019) for these critical values in closed forms). Normally, $\tau ^R_*<\tau ^D_*$. We will concentrate the case where there is a positive mobility ${{\bar{m}}}_a$ and the delay is within $[\tau ^R_*, \tau ^D_*]$, so tick population in patch R alone will converge to the positive equilibrium, while tick populations in patch D exhibit oscillatory patterns as a local Hopf bifurcation of periodic solutions takes place near the positive equilibrium.

When ${{\bar{m}}}_a$ starts to increase from 0, two patches are connected by host mobility and system (31) becomes a transcendental function involving $\sin (n\omega \tau )$ and $\cos (n\omega \tau )$ with $n=1,2,3$. We look for solutions of $(\omega , \tau )$ as a continuous function from $(\omega ^D_*, \tau ^D_*)$ with $\delta$ as the varying parameter. However, a combination of the lack of a close form of the equilibrium as a function of the parameters including the delay and the mobility rate, the model as a system rather than a scalar equation and the existence of multiple delays makes the Hopf bifurcation analysis so complicated that the analytic calculation for the critical value and the Lyapunov coefficient of a Hopf bifurcation of periodic solution is not feasible. We will design some numerical simulations in next section based on the estimated critical values of delay and mobility from a continuation procedure.

## Numerical Simulation

In this study, we consider the homogeneous situation, so seasonal variations are incorporated by taking the unit time as one year to normalize the parameter values taken from some published literature. We refer to the monograph Zhang and Wu (2020) for the original sources of the parameter values:

$$\begin{aligned} \rho _{epa}^R= & {} 0.495; \rho _{efa}^R=0.435; \rho _{efn}^R=0.44; \rho _{efl}^R=0.28;\\ p^R= & {} p^D=2000; s^R=s^D=0.01; \mu ^R=\mu ^D=1; \delta =0.5; \tau _{fa}=7/365. \end{aligned}$$

We also fix all proportions of development rates from one stage to the next at the same value $\kappa$, i.e.,

$$\begin{aligned} \kappa =\kappa _p=\kappa _a=\kappa _n=\kappa _l=0.55. \end{aligned}$$

**Fig. 5:**
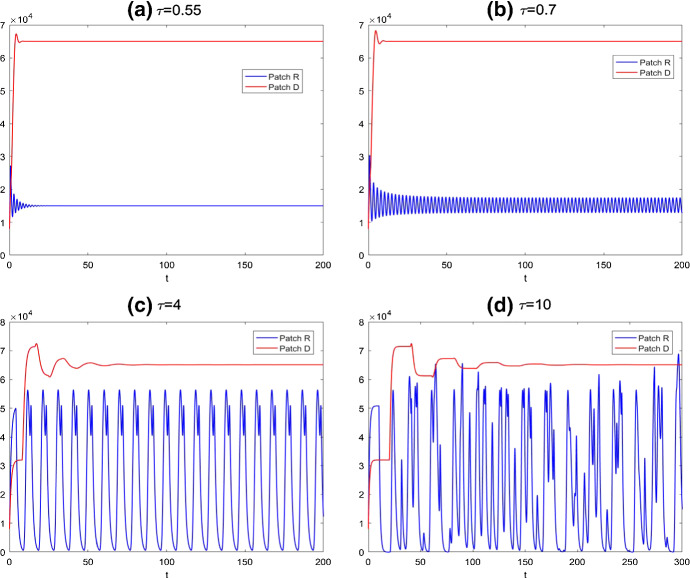
Solutions of egg density for the model (4) without host mobility between the two patches *R* and *D*, where blue line denotes the egg density in regular patch and the red line represents the one in diapause patch: **a** $\tau =0.55$; **b** $\tau =0.7$; **c** $\tau =4$; **d** $\tau =10$ (Color figure online)

**Fig. 6:**
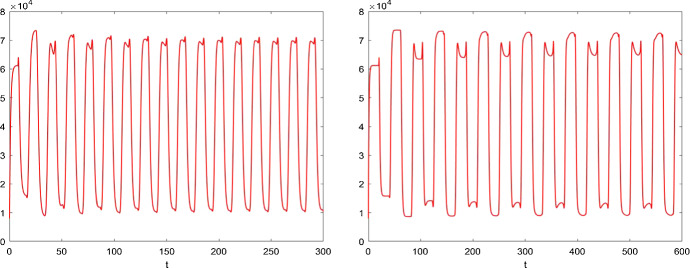
Compared with Fig. 5c and d, solutions of egg density for the model (4) for $\kappa =0.7$. Clearly, increasing $\kappa$ will contribute the oscillation phenomenon for egg density in the diapause patch

Without mobility, the model (4) has a positive equilibrium $E_*(14970, 65090)$. Considering the delay $\tau$ as the bifurcation parameter, we can calculate the critical value $\tau ^*=0.69$. From Fig. 5a, we observe that the egg density in regular patch $E^R(t)$ is locally asymptotically stable when $\tau =0.55$. The qualitative behavior of egg density $E^R(t)$ in model (4) undergoes periodic solution, dual-peak and more complex oscillation with increasing delay $\tau$, which is illustrated in Fig. 5: (b) $\tau =0.7$; (c) $\tau =4$; (d) $\tau =10$. However, the solutions of egg density in the diapause patch $E^D(t)$ do not change with different delays and remain stable. Note that when we increase the proportion $\kappa$ into 0.7, Fig. 6 compared with Fig. 5c and d shows that oscillation of egg density in the diapause patch starts to emerge. This shows that lower survival probability can keep egg dynamics in diapause patch to be stable.

**Fig. 7:**
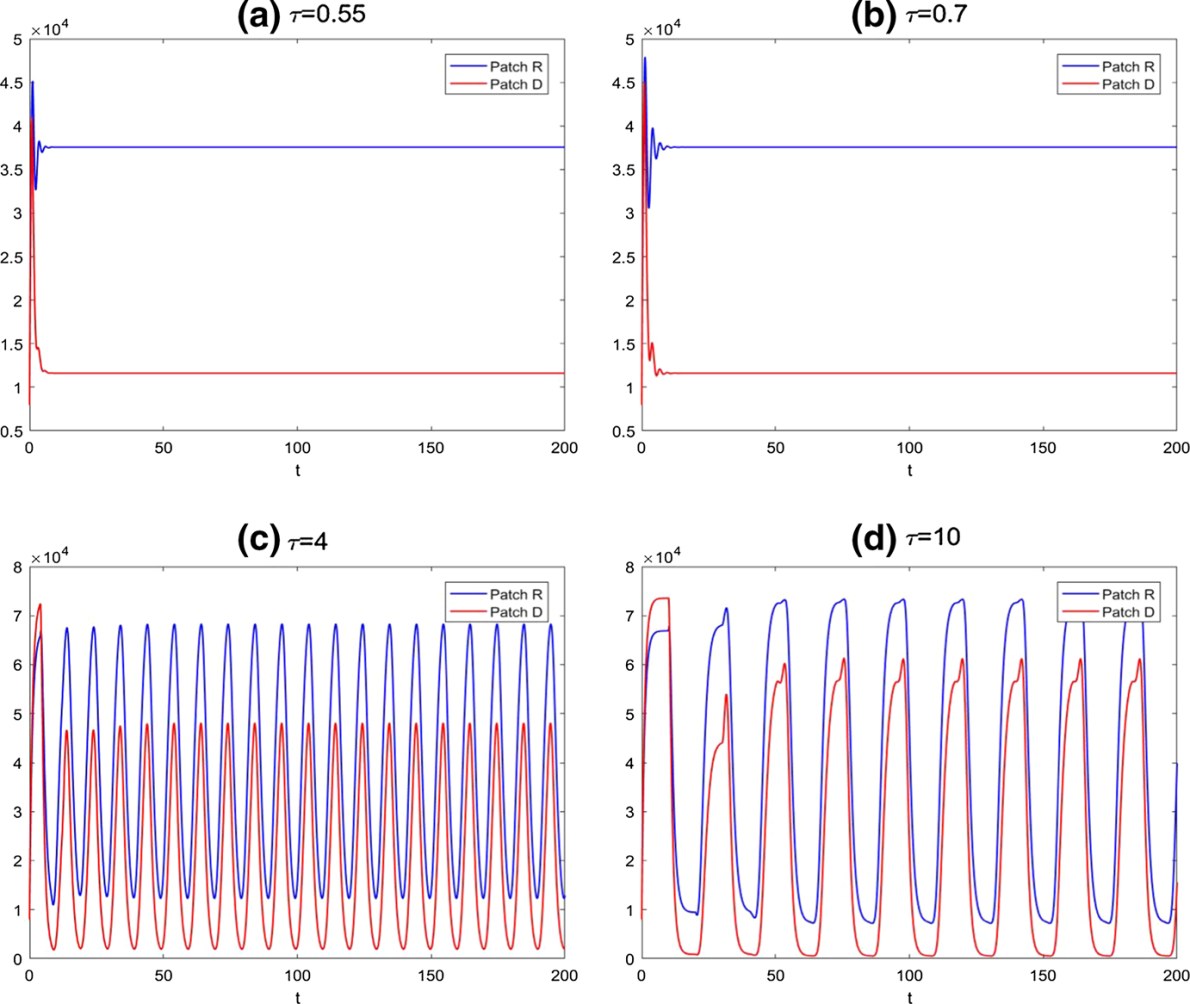
Solutions of egg density for the model (4) with host mobility between the two patches *R* and *D*, where blue line denotes the egg density in regular patch and the red line represents the one in diapause patch. We still choose the same time delays as those in Fig. 5: **a** $\tau =0.55$; **b** $\tau =0.7$; **c** $\tau =4$; **d** $\tau =10$ (Color figure online)

When the host movement is considered between patch R and patch D, and using the mobility ${\bar{m}}_a=146$ as an illustration, we show that feeding adult ticks will be carried over between the two patches and the positive equilibrium becomes (37,550, 11,620). Therefore, first of all, the host mobility sustains the persistence of tick population in the both patches, though the regular patch is the preferred habitat. This is illustrated in Fig. 7a and b.

**Fig. 8:**
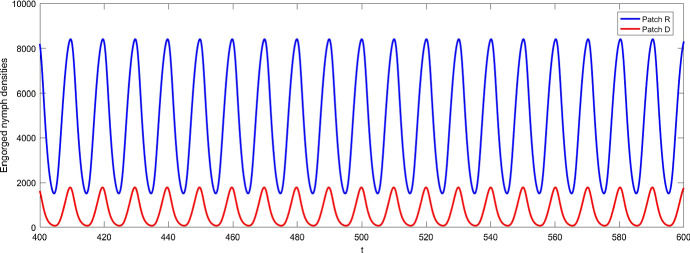
Solutions of engorged nymph densities for the model (4) with host mobility between the two patches *R* and *D* for $\tau =4$, where blue line denotes the nymph density in regular patch and the red line represents the one in diapause patch; other parameters are the same as those in Fig. 7 (Color figure online)

With the same value of delays as in Fig. 5, we describe the dynamics of model (4). From Fig. 7b, it is clear that the egg density is stable when $$\tau =0.7$$ and the critical value of time delay of model (4) with host movement is getting larger, $$\tau ^{**}=2.12$$. With increasing value of the time delay, the behaviors of egg densities in both patches become periodic, as shown in Fig. 7c and d. We also plot, when $$\tau =4$$, the engorged nymph densities in Fig. 8. We also compare Fig. 7b–d with Fig. 5b–d, and we note that egg densities in the regular patch are asymptotically constant with host mobilities when $$\tau =0.7$$ and exhibit much simpler single-peak oscillatory pattern when $$\tau =4$$ and $$\tau =10$$ in comparison with dual-peak oscillations and complex oscillation in Fig. 5b–d. Hence, host mobility not only synchronizes but also simplifies the collective behaviors.

In order to better understand the parameter values listed above, we normalize the model (4) with ${\tilde{E}}^R(t)=E^R(\dfrac{t}{365})$ and ${\tilde{E}}^D(t)=E^D(\dfrac{t}{365})$ and obtain

$$\begin{aligned} \begin{pmatrix}\dot{{\tilde{E}}}^R(t)\\ \dot{{\tilde{E}}}^D(t)\end{pmatrix} =\begin{pmatrix}\dfrac{1}{365}[f^R(q_{11}{\tilde{E}}^R(t-{\tilde{\tau }}^R)+q_{12}{\tilde{E}}^D(t-{\tilde{\tau }}^D))-\mu ^R{\tilde{E}}^R(t)]\\ \dfrac{1}{365}[f^D(q_{21}{\tilde{E}}^R(t-{\tilde{\tau }}^R)+q_{22}{\tilde{E}}^D(t-{\tilde{\tau }}^D))-\mu ^D{\tilde{E}}^D(t)]\end{pmatrix}, \end{aligned}$$

where ${\tilde{\tau }}^R=365\tau ^R$ and ${\tilde{\tau }}^D=365\tau ^D$; ${\tilde{E}}^R$ and ${\tilde{E}}^D$ represent the total numbers of eggs to be hatched within one day. Based on the process of normalization, it means that tick population will take $365\tau ^R$ or $365\tau ^D$ days in each patch to complete their life cycle from the view of biology. For instance, $\tau =0.55$ in Fig. 5a is equivalent to ${{\tilde{\tau }}}=0.55\times 365=200.75$, which implies that the life cycle of tick population in regular patch is 200.75 days.

## Discussion

It is commonly known that delay, if increased passing a critical value, in a negative feedback system destabilizes the system and generates nonlinear oscillations through the Hopf bifurcation mechanism. It is also known that increasing the delay further may lead to complicated oscillatory patterns including multi-peaks with a single period. Multiple and large delays may appear in tick population dynamics due to the so-called diapause development, when ticks suspend their development in response to unfavorable conditions. It was established in Shu et al. (2020) and Zhang and Wu (2019) that in single spatial location, when a portion of ticks undergo diapause during their life cycle, nonlinear oscillations can take place.

Here, we consider a landscape consisting of two patches (patch R and patch D) where ticks in different patches will experience regular (in patch R) and diapause (in patch D) development. When these are in isolation in terms of population dynamics of a particular tick species, we may observe patterns of stabilization to a positive equilibrium in one patch and oscillation in another patch with potentially multi-peaks within a given period. We then show the mobility of hosts on which the adult ticks feed for development can redistribute engorged adult ticks in two patches, creating two cohorts of ticks developing in two different habitats until they feed on hosts during the questing activities in the adult stage. The tick population dynamics is then described by a couple system of delay differential equations with two delays, the normal and diapause development delays. We show that the model system may have a unique positive equilibrium and its stability may change due to the mobility of the hosts and the diapause delay in combination. This will tend to synchronize the system to periodic oscillations with single peak within a given period. This synchronization to a simple-looking periodic solution seems to be new and may offer another angle to view tick population dynamics in the natural environment. It remains for further studies to see how seasonal variation of the habitat conditions coupled with the diapause delay can impact the tick population dynamics (this requires a periodic system of delay differential equations with time-dependent delays) and influence the patterns of tick-borne disease spread.
